# Incidence, clinical course, and risk factors in the development of femoral pseudoaneurysm after atrial fibrillation ablation

**DOI:** 10.1002/joa3.12950

**Published:** 2023-11-08

**Authors:** Takafumi Koyama, Kazuki Tobita, Tatsuto Kawaguchi, Shuhei Uchida, Eiji Koyama, Nobuhisa Kodera, Yusuke Tamaki, Yuri Otomaru, Hirokazu Miyashita, Takayoshi Yamashita, Shingo Mizuno, Masato Murakami, Shigeru Saito

**Affiliations:** ^1^ Department of Cardiology Shonan Kamakura General Hospital Kamakura Japan

**Keywords:** aneurysm false*/etiology, atrial fibrillation, catheter ablation/adverse effects*, thrombin*

## Abstract

**Background:**

Previous studies have revealed the risk factors for femoral pseudoaneurysms (FPA). Most data on FPA are based on coronary and peripheral interventions, with limited studies focusing on atrial fibrillation (AF) ablation. However, patient backgrounds, anticoagulation regimens, and vascular access methods differ. In addition, a standard for managing FPA after AF ablation remains elusive due to the difficult nature of achieving thrombosis in pseudoaneurysms.

**Methods:**

This single‐center, retrospective, observational study included 2805 consecutive patients who underwent AF ablation between January 2016 and December 2021. All patients underwent femoral artery and vein punctures. Puncture sites were checked 1 day post‐procedure.

**Results:**

A total of 23 FPA patients were identified during the study period. Multivariate logistic regression analysis showed that hypertension (odds ratio 4.66, 95% confidence interval: 1.38–15.71; *p* = .0032) and warfarin use (odds ratio 3.83, 95% confidence interval: 1.40–10.45; *p* = .021) were significantly associated with the occurrence of FPA. The compression success rate was low (22%). There were nine and six patients in the endovascular treatment (EVT) and ultrasound‐guided thrombin injection (UGTI) groups, respectively. The success rates were 100% and 84% in the EVT and UGTI groups, respectively. The length of hospital stay after FPA treatment was 2.1 days in the EVT group and 1.3 days in the thrombin group.

**Conclusion:**

We must be careful about post‐procedural FPA, especially for hypertension and warfarin‐using patients. Treatment of pseudoaneurysms with anticoagulants is unlikely to achieve hemostasis, and an early switch to invasive treatments, such as EVT, should be considered.

## Graphical abstract

In our investigation of atrial fibrillation ablation, we identified warfarin and hypertension as primary risk factors for femoral pseudoaneurysms. Our findings indicated that endovascular balloon inflation and thrombin injection were effective, with minimal complications and shortened hospital stays.

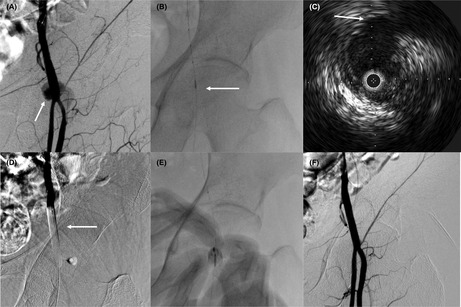

## 1 INTRODUCTION

Atrial fibrillation (AF) ablation is performed worldwide, with the number of AF ablations performed seeing a significant increase over the last decade.^1^ Due to the use of multiple sheaths and the need for anticoagulation during and after ablation, vascular complications are common adverse events associated with AF ablation. One of these complications includes femoral pseudoaneurysms (FPA), which has a reported incidence of 0.53%–0.93%.^2^

Previous studies have reported on the risk factors for FPA, such as older age, obesity, and hypertension.^3^ However, most of these are reported after coronary intervention and differ from AF ablation regarding patient background, anticoagulation regimen, and vascular access. In addition, a standardized treatment of FPA with anticoagulant therapy remains undefined. Hence, this study aimed to investigate the risk factors, incidence, and treatment of pseudoaneurysms after AF ablation.

## 2 METHODS

### 2.1 Study population

Between January 2016 and December 2021, 2806 consecutive patients who underwent AF ablation at Shonan Kamakura General Hospital were included in this single‐center, retrospective, observational study. The hospital's Ethics Committee approved this retrospective study, which did not require written informed consent.

Patient data and clinical outcomes were obtained from medical records. The surgeons examined the puncture site the day after the procedure. In cases where findings such as tenderness, vascular murmur, or swelling were confirmed, Doppler sonography was performed to diagnose a pseudoaneurysm.

### 2.2 Anticoagulation therapy

All patients underwent anticoagulation therapy using either warfarin or a direct oral anticoagulant (DOAC) for at least 3 weeks prior to catheter ablation, regardless of their CHADS2 score or the presence or absence of sinus rhythm. While most patients took DOACs as their anticoagulation therapy, warfarin was the preferred choice in cases of severely compromised renal function or when the patient was undergoing dialysis. For those who were administered warfarin before the procedure, the international normalized ratio was consistently maintained within a range of 1.6–2.6.^4^ Anticoagulation with both DOAC and warfarin was uninterrupted during the periprocedural period. Heparinized saline was infused to maintain an activated clotting time of at least 300 s.

### 2.3 Vascular access

Vascular access was obtained using the standard landmarks and palpations of the femoral artery pulse. For cryoballoon ablation, two femoral vein punctures were performed, introducing a 6 Fr sheath and a 15 Fr sheath (FlexCath Advance, Medtronic, Dublin, Ireland). In radiofrequency ablation, three femoral vein punctures were performed with the introduction of 8, 8.5, and 6 Fr sheaths. All patients underwent arterial puncture for blood pressure monitoring, utilizing a 3 Fr sheath in the femoral artery. Preoperative computed tomography evaluated the coronary arteries; should stenosis be present, a 4 Fr sheath was inserted into the femoral artery to facilitate coronary angiography.

### 2.4 Ablation procedures

The procedure was performed under moderate propofol sedation. Pulmonary vein isolation was performed using either a 28‐mm cryoballoon (Arctic Front Advance, Medtronic, Ireland), an 8‐mm tip ablation catheter (Japan Lifeline, Inc., Tokyo, Japan), TactiCathTM Quartz (Abbott, Ravenswood, Chicago, IL, USA), or a SmartTouch ThermoCool Surround Flow irrigated‐tip ablation catheter (Biosense Webster, Irvine, CA, USA). After the procedure, hemostasis of the vascular entry site was achieved using manual compression after intravenous injection of protamine and figure‐of‐eight sutures.

### 2.5 Treatment strategy for pseudoaneurysms

Anticoagulation therapy continued after confirmation of FPA because the damaged endocardium and low atrial contraction might cause thrombotic complication. If there was only severe anemia, we temporarily stopped anticoagulants.

All patients initially underwent conventional manual or ultrasound‐guided compression therapy (UGCT). The treating physician determined the appropriate timing for transitioning from manual compression, or UGCT, to thrombin injection, considering patient tolerance and therapeutic efficacy. Before 2019, the primary modality for treating compression‐resistant pseudoaneurysms was ultrasound‐guided thrombin injection (UGTI). Surgical intervention was selected in cases where the pseudoaneurysm was large or accompanied by an arteriovenous fistula. After 2019, we refined our protocol to incorporate combined endovascular treatment (EVT) with percutaneous thrombin injection as the first‐line therapy for pseudoaneurysms unresponsive to compression.

### 2.6 Echo‐guided thrombin injection

Treatment was administered using either convex or linear transducers, selected between 5 and 7.5 MHz based on the depth of the pseudoaneurysm. Prior to injection, the site was meticulously prepared, and a local anesthetic, typically 1%–2% lidocaine, was applied to both the skin and underlying tissue. A solution containing 1000 IU/mL thrombin was prepared by combining 5000 IU thrombin with 5 mL of normal saline. This solution was transferred to a 1 mL syringe and introduced under the precise guidance of ultrasound imaging. A 21‐gauge needle was carefully inserted adjacent to the pseudoaneurysm sac in the neck region, followed by the deliberate injection of thrombin solution into the sac. The utmost caution was exercised to ensure that the thrombus remained confined within the sac. Thrombin was consistently administered until the pseudoaneurysm was nearly entirely thrombosed, accompanied by the disappearance of color Doppler flow within the sac. After needle withdrawal from the pseudoaneurysm, an ultrasound assessment was performed to verify the absence of thrombosis around the originating artery. Following this procedure, all patients were instructed to maintain a supine posture for a minimum of 12 h.

### 2.7 EVT procedure

**FIGURE 1:**
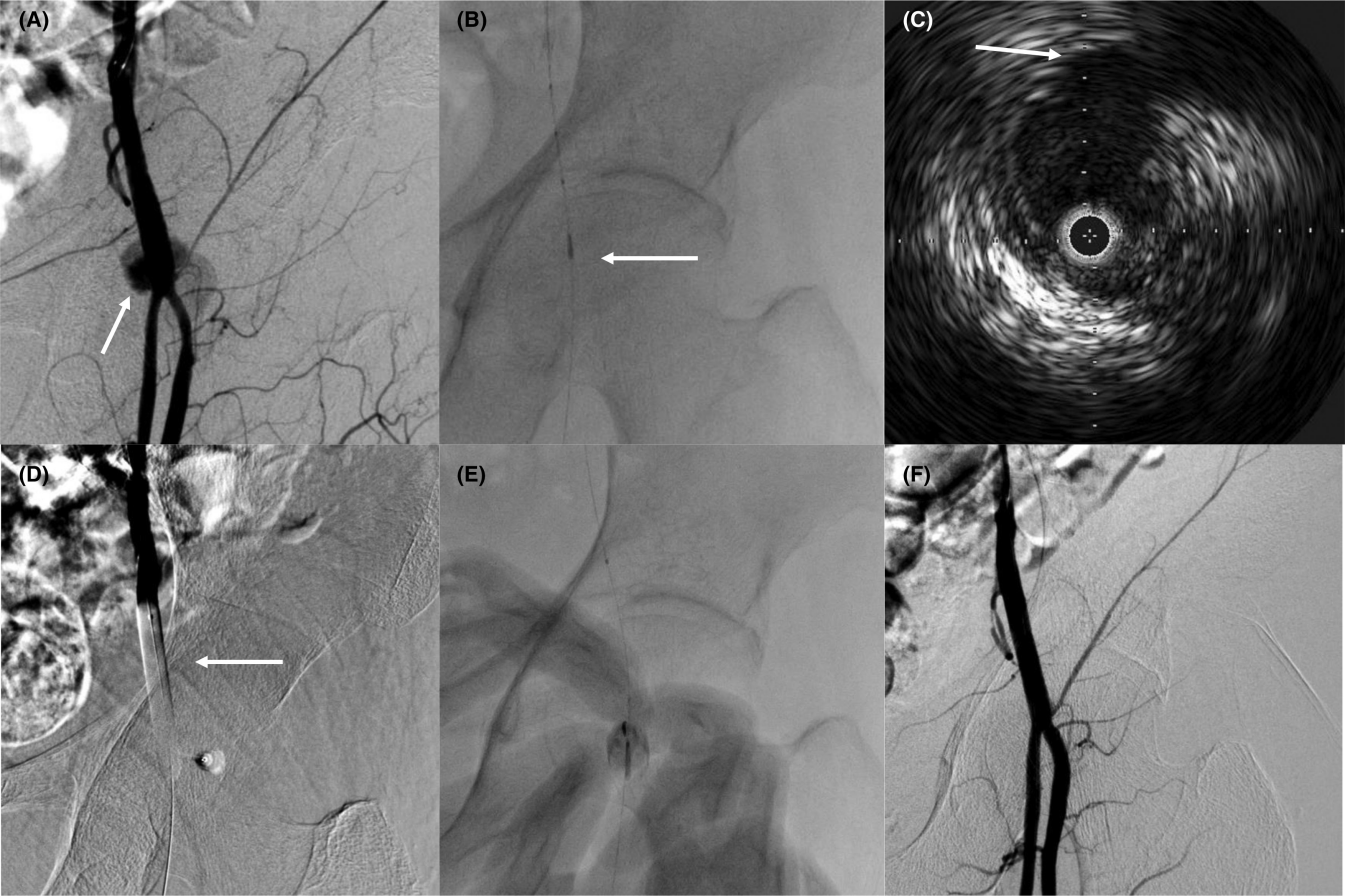
Angiographic imaging of a typical endovascular treatment process with percutaneous thrombin injection for a pseudoaneurysm of the femoral artery. (A) Angiography showing a pseudoaneurysm originating from the common femoral artery. The white arrow indicates the body of the pseudoaneurysm. (B) The guiding wire was advanced to the iliac artery from the right radial artery. (C) We used IVUS to measure the artery diameter. IVUS revealed a pseudoaneurysm in the common femoral artery. The white arrow indicates a pseudoaneurysm. (D) The balloon is inflated to achieve hemostasis inside the artery. Angiography showed that blood flow to the pseudoaneurysm was completely blocked. (E) Thrombin was injected into the pseudoaneurysm under fluoroscopic guidance. (F) The final angiogram showed complete hemostasis. IVUS, intravascular ultrasound.

A 4.5 or 6 Fr 120 cm guiding sheath was inserted into the radial artery. The guiding sheath was advanced into the external iliac artery, and angiography was performed to confirm the location of the pseudoaneurysm. A 0.014‐inch guidewire was advanced to the distal part of the superficial femoral artery (SFA), and the vessel diameter was confirmed by intravascular ultrasound examination. The balloon was then inflated to stop blood flow to the pseudoaneurysm. Next, thrombin was delivered to the aneurysm under fluoroscopic guidance. After balloon deflation, angiography confirmed the absence of blood flow into the pseudoaneurysm (Figure 1).

### 2.8 Statistical analysis

All statistical analyses were performed using JMP, version 14.0 (SAS Institute, Cary, NC, USA). Continuous data are expressed as means ± standard deviation and were compared using the Student's *t*‐test. Categorical variables were compared using the χ^2^ test. Parameters with statistically significant differences (less than .05 in the univariate analysis) were entered into a multiple logistic regression analysis to explore factors associated with FPA incidence.

## 3 RESULTS

The patient demographics are shown in Table 1. A total of 23 FPA cases were identified during the study period. The prevalence of hypertension, chronic kidney disease, and warfarin use was higher in the FPA group than in the non‐FPA group. No significant differences were observed between the two groups in terms of other clinical characteristics. Multivariate logistic regression analysis revealed that hypertension and warfarin use were significantly associated with FPA occurrence (Table 2).

**TABLE 1:** Patient clinical characteristics and risk factors in relation to femoral pseudoaneurysm.

Variable	Non‐FPA group (*n* = 2783)	FPA group (*n* = 23)	*p*‐Value
Age (years)	68.9 ± 10.7	71.9 ± 9.8	.18
Male sex, *n* (%)	1817 (65.3%)	15 (65.2%)	.99
Diabetes mellitus	479 (17.2%)	3 (13.0%)	.6
Hypertension	1620 (58.2%)	21 (87.0%)	.005
Dyslipidemia	1005 (36.1%)	6 (26.1%)	.32
BMI (kg/m^2^)	23.5 ± 4.1	24.3 ± 3.2	.36
LAD, mm	40.0 ± 10.6	41.0 ± 4.9	.64
LVEF, %	59.5 ± 10.9	57.2 ± 9.5	.31
CHADS_2_ score	1.30 ± 1.08	1.66 ± 1.12	.099
Antiplatelet therapy	279 (10.0%)	4 (17.9%)	.24
Chronic kidney disease	1428 (51.4%)	17 (73.9%)	.031
Hemodialysis	72 (2.6%)	2 (8.7%)	.067
Warfarin therapy	155 (5.6%)	4 (17.3%)	.015
PT‐INR	1.21 ± 0.37	1.35 ± 0.60	.095
APTT, s	36.2 ± 13.1	36.5 ± 7.5	.94
Sheath size (femoral artery)			
3 Fr	2204 (79.2%)	16 (69.6%)	.26
4 Fr	579 (20.8%)	7 (30.4%)	

Abbreviations: APTT, activated partial thromboplastin time; BMI, body mass index; FPA, femoral pseudoaneurysm; INR, international normalized ratio; LAD, left atrial diameter; LVEF, left ventricle ejection fraction; PT, prothrombin time.

**TABLE 2:** Univariate and multivariate predictors of femoral pseudoaneurysm.

Factors	Univariate analysis			Multivariate analysis		
	OR	95% CI	*p*‐Value	OR	95% CI	*p*‐Value
Age	1.03	0.99–1.08	.13			
Male sex	1.07	0.45–2.50	.88			
Hypertension	5.02	1.49–16.86	.0018	4.66	1.38–15.71	.0032
BMI, kg/m^2^	1.03	0.97–1.09	.46			
LAD, mm	1.00	0.98–1.03	.74			
LVEF, %	0.98	0.95–1.02	.33			
CHADS_2_ score	1.35	0.96–1.88	.092			
Chronic kidney disease	2.82	1.11–7.12	.019			
Hemodialysis	3.44	0.80–14.92	.16			
Warfarin therapy	4.46	1.64–12.10	.011	3.83	1.40–10.45	.02
Sheath size	1.66	0.68–4.07	.28			

Abbreviations: BMI, body mass index; CI, confidence interval; LAD, left atrial diameter; LVEF, left ventricle ejection fraction; OR, odds ratio.

**FIGURE 2:**
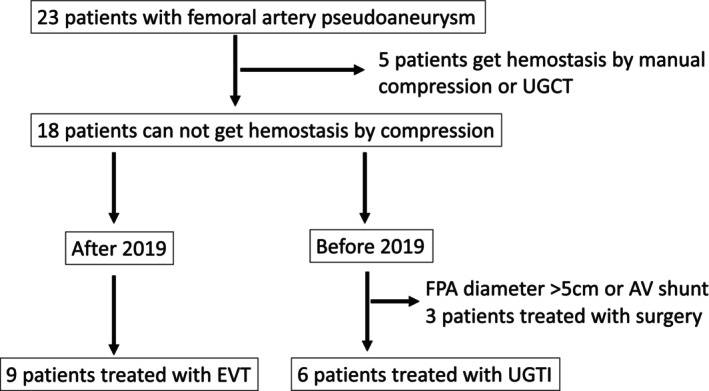
Flowchart of the modalities and outcomes of the treatment of 23 pseudoaneurysms. EVT, endovascular treatment; FPA, femoral pseudoaneurysm; UGCT, ultrasound‐guided compression therapy; UGTI, ultrasound‐guided thrombin injection.

In 23 patients, FPA was initially treated with manual compression, or UGCT, which was successful in five patients (22%). Before 2019, surgical procedures were conducted on three patients: two exhibited a diameter exceeding 50 mm, while the other presented a 9 mm diameter concomitant with an arteriovenous fistula. For the other six pseudoaneurysms that were unresponsive to compression, UGTI was selected for treatment. After 2019, nine cases of pseudoaneurysms unresponsive to compression were treated with EVT (Figure 2).

Table 3 shows the clinical outcomes associated with FPA. One case was unsuccessfully treated with UGTI and subsequently underwent surgical intervention. The success rates of EVT and surgery were 100%. No complications were observed in any of the treatment modalities. The average diameter of the pseudoaneurysms in the invasively treated groups was 30–40 mm. The average hospitalization duration after FPA treatment was 2.1 days for the EVT group, 1.3 days for the UGTI, and 4.7 days for the surgical group. It is evident that the surgical group had the longest hospitalization. Some pseudoaneurysms originate from both the SFA and the deep femoral artery (DFA).

**TABLE 3:** Aneurysmal risk characteristics in relation to the treatment of femoral pseudoaneurysm.

Characteristics	EVT (*n* = 9)	UGTI (*n* = 6)	Surgery (*n* = 3)	Compression (*n* = 5)
Success rate (%)	100	84	100	22
Complication rate (%)	0	0	0	0
Length of stay in hospital after FPA treatment (days)	2.1 ± 2.4	1.3 ± 0.8	4.7 ± 3.1	2.2 ± 2.2
Diameter of FPA (mm)	31.0 ± 20.0	37.0 ± 17.0	40.0 ± 27.0	18.0 ± 4.3
Time from puncture to treatment (days)	2.0 ± 1.7	6.2 ± 9.6	13.3 ± 15.0	1.0 ± 0.0
Location of the artery where FPA originates				
CFA (%)	22	50	33	60
SFA or DFA (%)	78	50	67	40

Abbreviations: CFA, common femoral artery; DFA, deep femoral artery; EVT, endovascular treatment; FPA, femoral pseudoaneurysm; SFA, superficial femoral artery; UGTI, ultrasound‐guided thrombin injection.

## 4 DISCUSSION

The present study demonstrates the clinical experience of FPA in a large cohort of patients. The main findings of the study were as follows: (a) the incidence of FPA after AF ablation was 0.82%, (b) hypertension and warfarin therapy were associated with a higher incidence of FPA, (c) the manual compression success rate for FPA was only 22%, and (d) the treatment outcomes of UGTI, EVT, and surgery were favorable.

Several patient and procedural characteristics predict FPA formation following percutaneous coronary intervention (PCI).^3^ However, these predictors have not been studied in post‐AF ablation populations. A valid comparison between the predictors of FPA and those complicating PCI is difficult because the patient characteristics, preoperative medications, and catheter tools are different. In multivariate analysis, we found that hypertension and warfarin use were independent risk factors for FPA formation. Hypertension elevates intravascular pressure and causes continuous bleeding at the puncture point. It has been reported that patients with systolic blood pressure >180 mmHg during the procedure were eight times more likely to develop vascular complications than other groups.^5^ The use of antihypertensive drugs may help reduce systolic blood pressure during ablation, possibly reducing the incidence of vascular complications.

In our study, pseudoaneurysms were more likely to occur in patients taking warfarin than in those taking DOACs. The mechanism of increased FPA with warfarin involves the inhibition of several coagulation factors, whereas DOAC is a direct thrombin inhibitor. In addition, the half‐life of warfarin is longer than that of DOAC. Several randomized controlled trials have compared the perioperative administration of warfarin and DOAC for ablation; for instance, the RE‐CIRCUIT (Randomized Evaluation of Dabigatran Etexilate Compared to Warfarin in Pulmonary Vein Ablation: Assessment of an Uninterrupted Periprocedural Anticoagulation Strategy) trial showed a greater incidence of major bleeding events (such as cardiac tamponade and inguinal hematoma) in the warfarin group.^6^ In the VENTURE‐AF trial, which compared rivaroxaban with warfarin, bleeding events were comparable between the two groups.^7^ However, the limitations of the VENTURE‐AF trial were its small sample size (*n* = 248) and low number of events. These studies and the results of our study suggest that preoperative warfarin may be more likely than DOAC to cause pseudoaneurysms.

The factors contributing to pseudoaneurysms include not only patient characteristics and anticoagulation drugs but also the puncture technique employed and the puncture site chosen. Among the physicians in this study, 80% (2244 cases) were fellows, and 20% (561 cases) were attending physicians who performed vascular punctures. There was no notable difference in the incidence of pseudoaneurysms between fellows and attending physicians (0.75% vs. 1.5%; *p* = .10). Our study suggests that less experienced doctors do not necessarily experience a heightened occurrence of FPAs. The risk of femoral artery pseudoaneurysms increased when the puncture site was not in the common femoral artery but in the superficial, DFA, or the external iliac artery.^8^ Utilizing real‐time ultrasound‐guided puncture can help visualize the common femoral artery and determine the optimal route of access.^9^ In our study, some pseudoaneurysms originated from superficial or deep femoral arteries. Thus, the use of ultrasound‐guided punctures may reduce the prevalence of pseudoaneurysms.

Prior research has shown that the occurrence of pseudoaneurysms was significantly lower in trans‐radial approach than in trans‐femoral approach.^10^ Trans‐radial route might decrease hemorrhagic complication even in AF ablation procedure, but the trans‐femoral approach is the preferred and most commonly used access for mechanical circulatory support, such as percutaneous cardiopulmonary support.^8^ In our institution, the arterial pressure line is placed in the femoral artery to facilitate the immediate deployment of mechanical support should complications arise during ablation. If the risk of pseudoaneurysm is high, such as with hypertension or oral warfarin, placement of an arterial pressure line in the radial artery is also an option.

No guidelines have been established regarding the treatment of iatrogenic FPA. In 1991, Fellmeth introduced UGCT as a safe and noninvasive method to treat FPA.^11^ However, when anticoagulants are used, the success rate of hemostasis of the FPA by UGCT is reportedly 30%–73%.^12^ Moreover, FPA compression is a painful procedure that requires a long time to achieve hemostasis.

UGTI is a treatment option for FPA. The utility of UGTI has long been documented; however, technical failures can easily occur with wide‐necked FPAs. Complications of UGTI such as venous thrombosis, pulmonary embolism, and arterial thrombosis have been reported.^13^ Because the EVT method occludes blood flow into the aneurysm using a balloon until hemostasis is achieved, thrombosis is unlikely to occur, and treatment can be performed regardless of the morphology of the neck and aneurysm.^14^ Our study demonstrated the feasibility of EVT for treating FPAs through our high procedural success rate, lack of complications, and short hospital stays. As hemostasis of pseudoaneurysms is challenging with anticoagulants, we recommend switching to EVT as soon as possible if hemostasis is difficult to achieve with compression.

This study had several limitations. First, this was a retrospective, non‐randomized, single‐center study. A large‐scale, prospective, randomized trial is warranted to confirm the study outcomes. Second, not all punctures were performed under echo guidance. Recently, angiography and duplex ultrasound guidance have become common during puncture procedures. The puncture method may also affect the occurrence of pseudoaneurysms. Third, the number of FPA cases was small; therefore, regression analysis was limited. Fourth, treatment selection was not randomly allocated, and there could be a selection bias in the treatment choice. Randomization is warranted to confirm the effects of each treatment.

## 5 CONCLUSION

Therefore, treating the development of post‐AF ablation FPA should be completed carefully, especially in individuals with hypertension and warfarin users. FPA on oral anticoagulants makes achieving hemostasis difficult using compression. Because of this, we would encourage an early switch to EVT.

## AUTHOR CONTRIBUTIONS

Takafumi Koyama was responsible for the clinical design and conceptualization. Takafumi Koyama, Kazuki Tobita, and Masato Murakami were involved in the acquisition of clinical data. Takafumi Koyama, Kazuki Tobita, and Masato Murakami analyzed and interpreted the data, and Takafumi Koyama wrote the manuscript. All the authors have discussed, read, and approved the submission of this manuscript to the journal.

## CONFLICT OF INTEREST STATEMENT

Authors declare no conflict of interests for this article.

## ETHICS STATEMENT

Authorization for the use of case information and materials was obtained from the Institutional Review Board of Shonan Kamakura General Hospital on April 4, 2023 (Approval No. TGE02165‐024).

## DECLARATIONS

*Approval of the Research Protocol*: Authorization for the use of case information and materials was obtained from the Institutional Review Board of Shonan Kamakura General Hospital (TGE02165‐024). *Registry and the Registration No*: N/A. *Animal Studies*: N/A.

## Data Availability

The data that support the findings of this study are available from the corresponding author, TK, upon reasonable request.

## References

[1] Calkins H , Hindricks G , Cappato R , Kim YH , Saad EB , Aguinaga L , et al. 2017 HRS/EHRA/ECAS/APHRS/SOLAECE expert consensus statement on catheter and surgical ablation of atrial fibrillation: executive summary. J Interv Card Electrophysiol. 2017 Oct;50(1):1–55. 28914401 10.1007/s10840-017-0277-z PMC5633646

[2] Cappato R , Calkins H , Chen SA , Davies W , Iesaka Y , Kalman J , et al. Worldwide survey on the methods, efficacy, and safety of catheter ablation for human atrial fibrillation. Circulation. 2005 Mar 8;111(9):1100–1105. 15723973 10.1161/01.CIR.0000157153.30978.67

[3] Webber GW , Jang J , Gustavson S , Olin JW . Contemporary management of postcatheterization pseudoaneurysms. Circulation. 2007 May 22;115(20):2666–2674. 17515479 10.1161/CIRCULATIONAHA.106.681973

[4] Inoue H , Okumura K , Atarashi H , Yamashita T , Origasa H , Kumagai N , et al. Target international normalized ratio values for preventing thromboembolic and hemorrhagic events in Japanese patients with non‐valvular atrial fibrillation: results of the J‐RHYTHM Registry. Circ J. 2013;77(9):2264–2270. 23708863 10.1253/circj.cj-13-0290

[5] Al‐Momani MS , AbuRuz ME . Incidence and predictors of groin complications early after coronary artery intervention: a prospective observational study. BMC Nurs. 2019 Dec;18(1):24. 31297032 10.1186/s12912-019-0349-8 PMC6599377

[6] Calkins H , Willems S , Gerstenfeld EP , Verma A , Schilling R , Hohnloser SH , et al. Uninterrupted dabigatran versus warfarin for ablation in atrial fibrillation. N Engl J Med. 2017 Apr 27;376(17):1627–1636. 28317415 10.1056/NEJMoa1701005

[7] Cappato R , Marchlinski FE , Hohnloser SH , Naccarelli GV , Xiang J , Wilber DJ , et al. Uninterrupted rivaroxaban vs. uninterrupted vitamin K antagonists for catheter ablation in non‐valvular atrial fibrillation. Eur Heart J. 2015 Jul 21;36(28):1805–1811. 25975659 10.1093/eurheartj/ehv177 PMC4508487

[8] Sandoval Y , Burke MN , Lobo AS , Lips DL , Seto AH , Chavez I , et al. Contemporary arterial access in the cardiac catheterization laboratory. JACC Cardiovasc Interv. 2017 Nov;10(22):2233–2241. 29169493 10.1016/j.jcin.2017.08.058

[9] Pellegrino PL , Di Monaco A , Santoro F , Grimaldi M , D'Arienzo G , Casavecchia G , et al. Near zero vascular complications using echo‐guided puncture during catheter ablation of arrhythmias: a retrospective study and literature review. J Arrhythmia. 2022 Jun;38(3):395–399. 10.1002/joa3.12723 PMC9237317 35785379

[10] Brueck M , Bandorski D , Kramer W , Wieczorek M , Höltgen R , Tillmanns H . A randomized comparison of transradial versus transfemoral approach for coronary angiography and angioplasty. JACC Cardiovasc Interv. 2009 Nov;2(11):1047–1054. 19926042 10.1016/j.jcin.2009.07.016

[11] Fellmeth BD , Roberts AC , Bookstein JJ , Freischlag JA , Forsythe JR , Buckner NK , et al. Postangiographic femoral artery injuries: nonsurgical repair with US‐guided compression. Radiology. 1991 Mar;178(3):671–675. 1994400 10.1148/radiology.178.3.1994400

[12] Eisenberg L , Paulson EK , Kliewer MA , Hudson MP , DeLong DM , Carroll BA . Sonographically guided compression repair of pseudoaneurysms: further experience from a single institution. Am J Roentgenol. 1999 Dec;173(6):1567–1573. 10584803 10.2214/ajr.173.6.10584803

[13] Mohler ER , Mitchell ME , Carpenter JP , Strandness DE , Jaff MR , Beckman JA , et al. Therapeutic thrombin injection of pseudoaneurysms: a multicenter experience. Vasc Med. 2001 Nov;6(4):241–244. 11958390 10.1177/1358836x0100600407

[14] Hayakawa N , Kodera S , Miyauchi A , Hirano S , Sahashi S , Ishibashi N , et al. Effective treatment of iatrogenic femoral pseudoaneurysms by combined endovascular balloon inflation and percutaneous thrombin injection. Cardiovasc Interv Ther. 2022 Jan;37(1):158–166. 33576932 10.1007/s12928-021-00764-9

